# Inhibition of NF-kB/IL-6/JAK2/STAT3 Pathway and Epithelial-Mesenchymal Transition in Breast Cancer Cells by Azilsartan

**DOI:** 10.3390/molecules27227825

**Published:** 2022-11-13

**Authors:** Rania Alaaeldin, Fares E. M. Ali, Amany Abdlrehim Bekhit, Qing-Li Zhao, Moustafa Fathy

**Affiliations:** 1 Department of Biochemistry, Faculty of Pharmacy, Deraya University, Minia 61111, Egypt; 2 Department of Pharmacology and Toxicology, Faculty of Pharmacy, Al-Azhar University, Assiut Branch, Assiut 71524, Egypt; 3 Department of Biochemistry, Faculty of Pharmacy, Minia University, Minia 61519, Egypt; 4 Department of Radiology, Graduate School of Medicine and Pharmaceutical Sciences, University of Toyama, Toyama 930-0194, Japan; 5 Department of Regenerative Medicine, Graduate School of Medicine and Pharmaceutical Sciences, University of Toyama, Toyama 930-0194, Japan

**Keywords:** azilsartan, breast cancer, NF-kB, EMT, IL-6/JAK2/STAT3, SLUG, TWIST, SNAIL

## Abstract

Metastatic breast cancer is an incurable form of breast cancer that exhibits high levels of epithelial-mesenchymal transition (EMT) markers. Angiotensin II has been linked to various signaling pathways involved in tumor cell growth and metastasis. The aim of this study is to investigate, for the first time, the anti-proliferative activity of azilsartan, an angiotensin II receptor blocker, against breast cancer cell lines MCF-7 and MDA-MB-231 at the molecular level. Cell viability, cell cycle, apoptosis, colony formation, and cell migration assays were performed. RT-PCR and western blotting analysis were used to explain the molecular mechanism. Azilsartan significantly decreased the cancer cells survival, induced apoptosis and cell cycle arrest, and inhibited colony formation and cell migration abilities. Furthermore, azilsartan reduced the mRNA levels of *NF-kB, TWIST, SNAIL, SLUG* and *bcl2*, and increased the mRNA level of *bax*. Additionally, azilsartan inhibited the expression of IL-6, JAK2, STAT3, MMP9 and bcl2 proteins, and increased the expression of bax, c-PARP and cleaved caspase 3 protein. Interestingly, it reduced the in vivo metastatic capacity of MDA-MBA-231 breast cancer cells. In conclusion, the present study revealed, for the first time, the anti-proliferative, apoptotic, anti-migration and EMT inhibition activities of azilsartan against breast cancer cells through modulating NF-kB/IL-6/JAK2/STAT3/MMP9, TWIST/SNAIL/SLUG and apoptosis signaling pathways.

## 1. Introduction

Breast cancer is the most diagnosed type of cancer in women worldwide. Despite the decline in mortality rates of breast cancer, breast cancer remains the leading cause of cancer-related death in females around the globe [1]. The complexity of therapeutic approaches for breast cancer comes from the fact that breast cancer is a heterogenous disease with various genetic backgrounds [2]. Currently, clinical decisions are made based upon the expression of hormonal receptors, human epidermal growth factor receptor 2 (HER2), and the extent of the disease [3].

Metastatic breast cancer is an incurable form of breast cancer that exhibits high levels of epithelial-mesenchymal transition (EMT) markers, which results in acquiring high metastatic abilities and possessing cancer stem cells-like characteristics (CSCs) [4]. It was reported that metastatic breast cancer showed resistance to currently available therapeutic strategies, including chemotherapy, radiotherapy, surgery, endocrine therapy and targeted biological therapy [5].

Angiotensin II (Ang II) is a biological active member of renin-angiotensin system (RAS) that shows potent vasoconstriction activity with the ability to regulate body fluid hemostasis and blood pressure [6]. In addition to the well-known physiological role of Ang II in the renal and cardiovascular systems, it has been linked to various signaling pathways involved in tumor cell growth and metastasis [7,8,9,10]. It was reported that Ang II downstream signaling leads to the activation of nuclear factor-kappa B (NF-kB) [11], which is a transcription factor involved in many cellular pathways and also a key regulator of different genes expression in malignant tumors [12,13]. Activation of NF-kB leads to the activation of interleukin-6 (IL-6), an inflammatory cytokine, with further activation of the downstream signals [14,15,16,17].

Signal transducer and activator of transcription (STAT) family is one of the most prominent transcription factors families that play an integrating role in the malignancy, progression, invasion, metastasis and chemoresistance of breast cancer. Furthermore, upstream regulators and downstream targets of STAT family have been reported to be activated in breast cancer diseases [18]. Janus kinase 2 (JAK2) is one of the upstream regulators of STAT3, and the JAK2/STAT3 pathway has been shown to be affected by the expression levels of activating ligands, like IL-6 [19].

Inspecting new therapeutic applications for the natural [20,21,22] or the synthetic agents [23,24,25] has become a great concern [26,27,28,29]. Thus, in the present study, we aimed to investigate, for the first time, the anti-proliferative activity and the effect on EMT process of azilsartan, which is an Ang II receptor blocker (ARB) [30], against breast cancer cell lines MCF-7 and MDA-MB-231 at the molecular level through examining the NF-kB/IL-6/JAK2/STAT3, TWIST/SNAIL/SLUG and apoptosis signaling pathways.

## 2. Results

### 2.1. Cell Viability Assay

**Figure 1:**
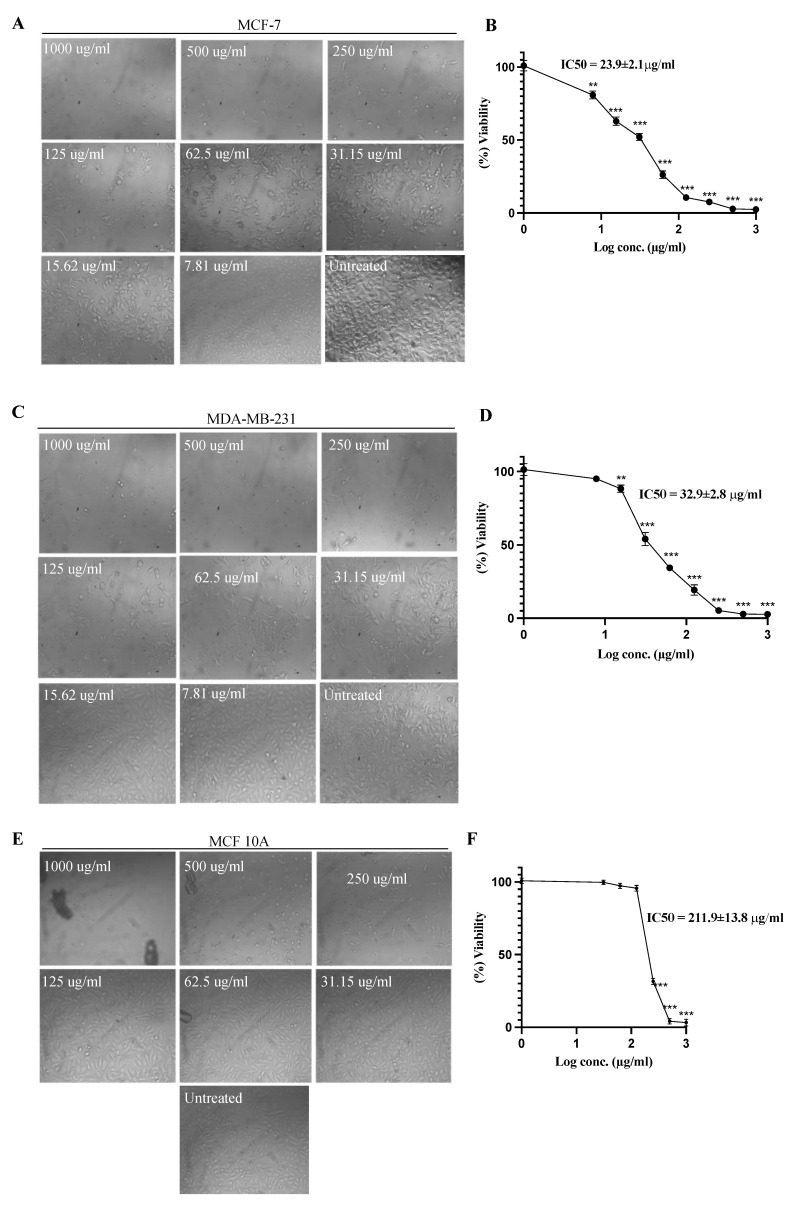
Effect of azilsartan on the viability of MCF-7 and MDA-MB-231 breast cancer cell lines and MCF 10A breast normal epithelial cells. (**A**,**C**,**E**) Representative images of the MCF-7, MDA-MB-231 and MCF 10A cell lines, respectively, before and after treatment with azilsartan at different concentrations for 48 h. (**B**,**D**,**F**) Percentage of viability of MCF-7, MDA-MB-231 and MCF 10A cell lines, respectively, after treatment with azilsartan for 48 h at different concentrations. Data represent mean ± SD, *n* = 3. Significant difference was analyzed by one-way ANOVA, where: ** *p* < 0.01, *** *p* < 0.001, compared to untreated cells.

The effect of azilsartan was tested at different concentrations against the survival of MCF-7 and MDA-MB-231 breast cancer cells. Survival of the cells was expressed as a percentage relative to that of the untreated cells, which was considered 100%. As shown in Figure 1, azilsartan significantly (*p* < 0.001) inhibited the proliferation of both cancer cells in a dose-dependent manner. The IC_50_ of azilsartan was 23.9 ± 2.1 μg/mL and 32.9 ± 2.8 μg/mL for MCF-7 and MDA-MB-231 cancer cell lines, respectively. Additionally, the effect of azilsartan was examined against MCF 10A breast epithelial normal cells at different concentrations, whereas the IC50 was obtained at a higher concentration at 211.9 ± 13.8 μg/mL, suggesting the safety of azilsartan.

### 2.2. Annexin V Analysis

**Figure 2:**
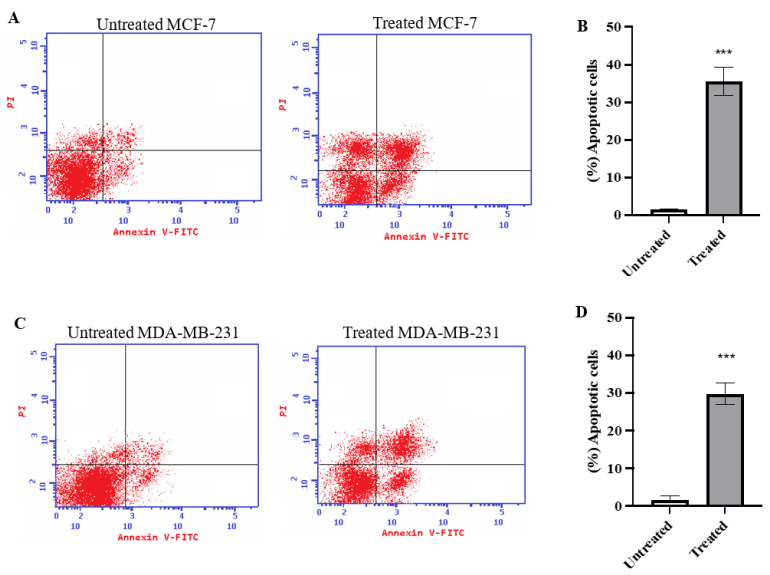
Effect of azilsartan on apoptosis of MCF-7 and MDA-MB-231 breast cancer cell lines estimated with Annexin V staining. (**A**,**C**) Representative dot plots for MCF-7 and MDA-MB-231 breast cancer cell lines, respectively. (**B**,**D**) The percentage of total apoptotic cells in MCF-7 and MDA-MB-231 breast cancer cell lines, respectively, after treatment with the IC_50_ of azilsartan for 48 h. Data represent mean ± SD, *n* =3. Significant difference was analyzed by one-way ANOVA test, where *** *p* < 0.001, compared to untreated cells.

To evaluate the percentage of total apoptosis exerted by azilsartan treatment on MCF-7 and MDA-MB-231 cancer cells for 48 h, annexin V assay was performed. As shown in Figure 2A,B, the percentage of total apoptotic cells in MCF-7 cancer cell line treated with azilsartan significantly increased (*p* < 0.001) from 1.62% ± 0.10 (in untreated cells) to 35.54% ± 3.8 (in treated cells). Regarding MDA-MB-231 cancer cell line, the percentage of apoptotic cells after treatment with azilsartan significantly increased (*p* < 0.001) from 1.69% ± 0.93 (in untreated cells) to 29.74% ± 2.80 (in treated cells), as shown in Figure 2C,D.

### 2.3. Cell Cycle Analysis Using Flowcytometry

**Figure 3:**
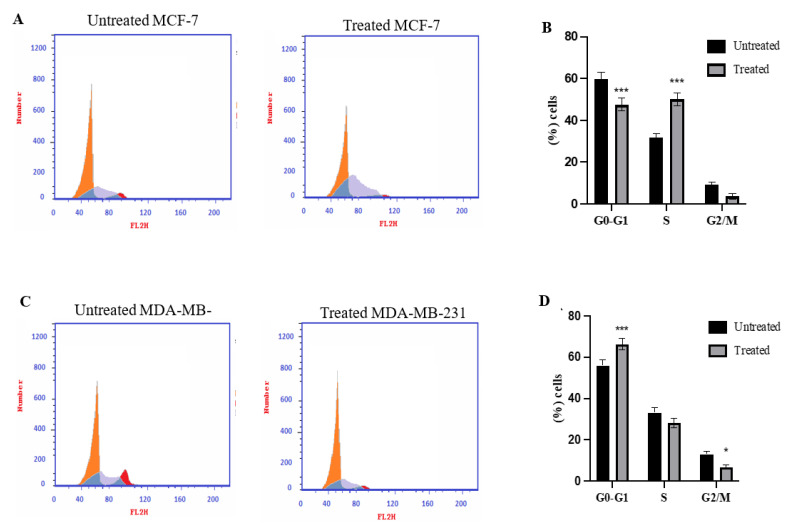
Effect of azilsartan on cell cycle phase distribution of MCF-7 and MDA-MB-231 breast cancer cell lines determined by flow cytometric analysis. (**A**,**C**) Representative histograms for MCF-7 and MDA-MB-231 breast cancer cell lines, respectively, after PI staining. (**B**,**D**) Percentage of cells in the various cell cycle phases in MCF-7 and MDA-MB-231 breast cancer cell lines, respectively. Data represent mean ± SD, *n* = 3. Significant difference was analyzed by Two-way ANOVA test, where * *p* < 0.05, *** *p* < 0.001 compared to untreated cells.

The cell cycle distribution of MCF-7 and MDA-MB-231 cancer cells before and after treatment of azilsartan was estimated using flowcytometry. As shown in Figure 3A,B, the percentage of MCF-7 cancer cells treated with azilsartan in G1 phase was significantly decreased (*p* < 0.001) to 47.48% ± 3.07 and in S phase was significantly increased (*p* < 0.001) to 49.95% ± 3.09, compared to the untreated cells. While MDA-MB-231 cancer cells treated with azilsartan showed a significant increase (*p* < 0.001) in G1 phase to 66.25% ± 2.74 and a significant decrease (*p* < 0.05) in G2/M phase to 6.78% ± 1.10, compared to untreated cells, as shown in Figure 3C,D.

### 2.4. Colony Formation Assay

**Figure 4:**
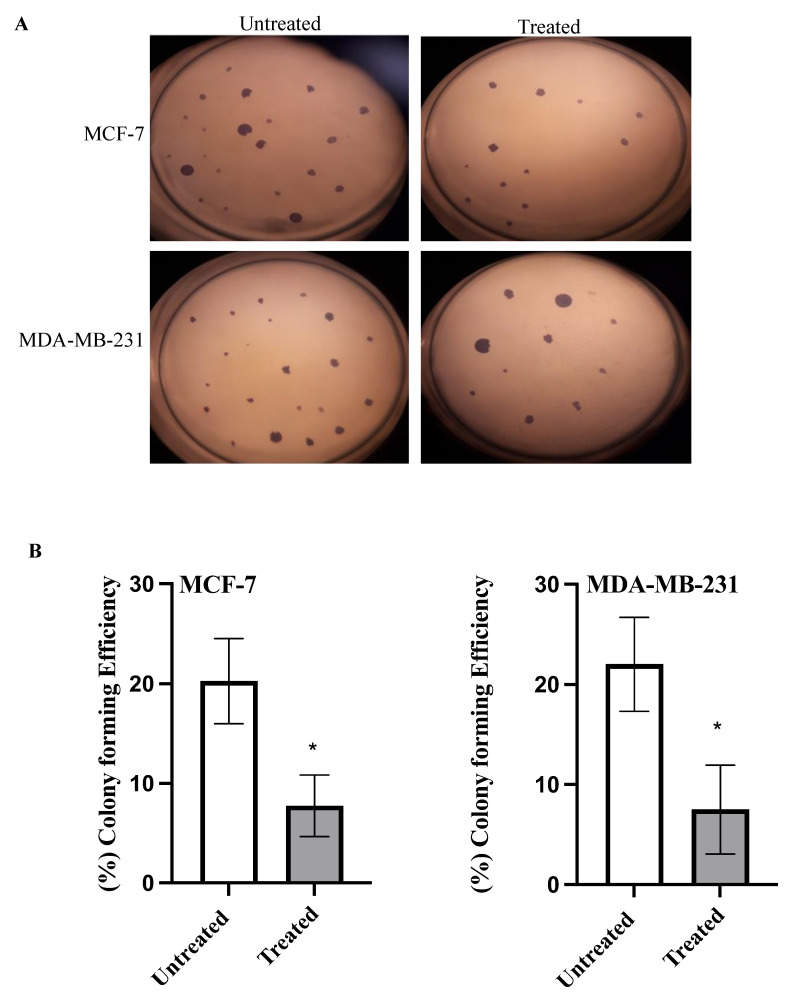
Effect of azilsartan on colony formation ability of MCF-7 and MDA-MB-231 breast cancer cell lines. (**A**) Representative images for the colonies formed before and after treatment with the IC_50_ of azilsartan in MCF-7 and MDA-MB-231 cell lines. (**B**) The percentage of colony forming efficiency of both cancer cell lines. Bars represent mean ± SD, *n* = 3. Significant difference was analyzed using student *t* test, where * *p* < 0.05, compared to untreated cells.

To investigate the ability of azilsartan to inhibit colonization, colony formation assay was performed. As shown in Figure 4, azilsartan significantly (*p* < 0.05) decreased colony forming efficiency in both MCF-7 and MDA-MB-231 cancer cells to 7.75% ± 3.09 and 7.5% ± 4.43, respectively, compared to untreated cells.

### 2.5. Cell Migration Assay

**Figure 5:**
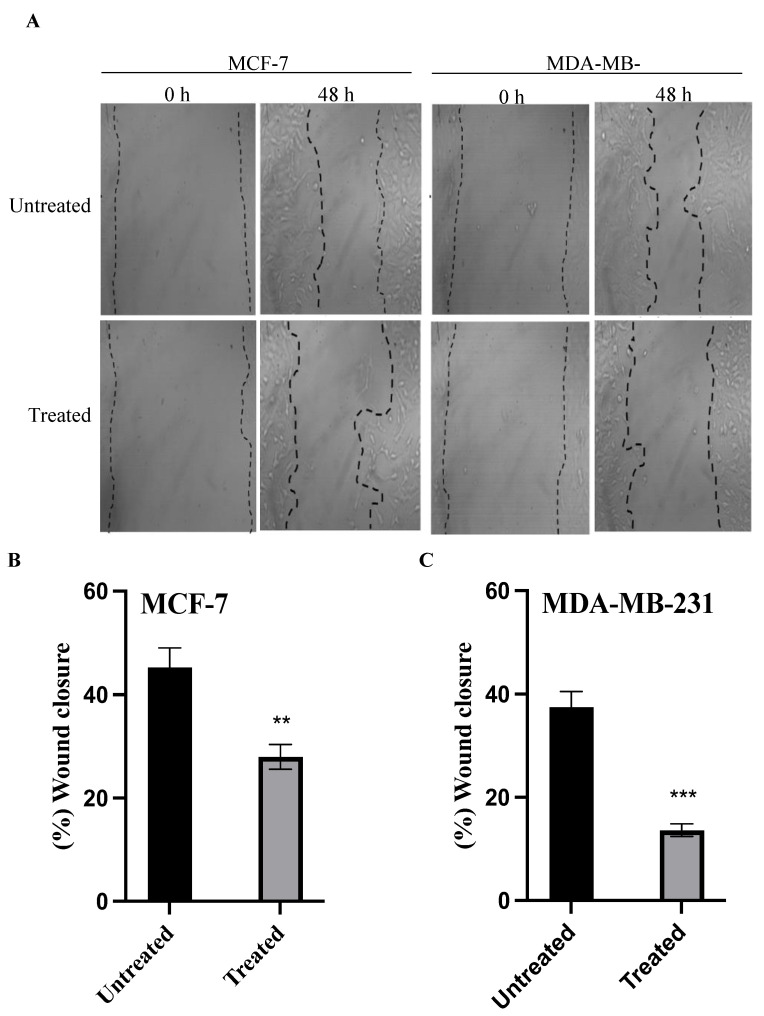
The effect of azilsartan on the migration ability of MCF-7 and MDA-MB-231 breast cancer cell lines. (**A**) Representative photos taken at 0 and 48 h for untreated and treated cells with the IC_50_ of azilsartan after making the scratch area for MCF-7 and MDA-MB-231 cancer cells. Magnification: ×40. (**B**,**C**) Percentage of the wound closure for MCF-7 and MDA-MB-231 cancer cell lines, respectively. Data represent mean ± SD, *n* = 3. Significant difference was analyzed by student *t* test, where ** *p* < 0.01, *** *p* < 0.001, compared to untreated cells.

The efficacy of azilsartan to inhibit the migration ability of MCF-7 and MDA-MB-231 cancer cells was evaluated by the scratch assay. Azilsartan was shown to significantly inhibit (*p* < 0.01) the scratch closure area to 27.97% ± 2.4 in treated MCF-7 cancer cells, compared to untreated cells. Azilsartan significantly (*p* < 0.001) decreased the wound closure area to 13.64% ± 1.21 in treated MDA-MB-231 cancer cells when compared to untreated cells, as shown in Figure 5.

### 2.6. Effect of Azilsartan on the Levels of Bax, Bcl2, Cleaved PARP Proteins

**Figure 6:**
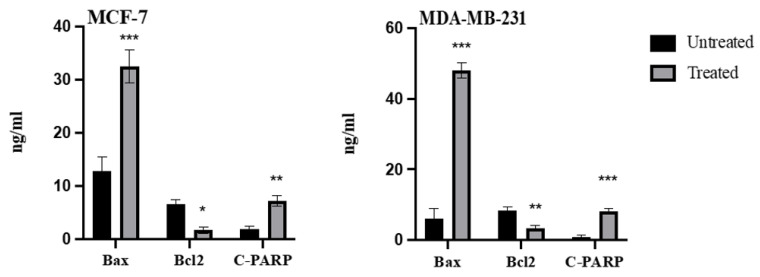
Effect of azilsartan on the levels of bax, bcl2 and c-PARP proteins. Bars represent mean ± SD, *n* = 3. Significant difference was analyzed by two-way ANOVA, where * *p* < 0.05, ** *p* < 0.01, *** *p* < 0.001, compared to untreated cells.

To confirm the initiation of apoptotic pathways, levels of bax, bcl2 and cleaved poly-ADP-ribose polymerases (c-PARP) proteins were measured before and after treatment with the IC50 of azilsartan. As shown in Figure 6, bax protein levels were significantly (*p* < 0.001) increased after treatment with azilsartan in MCF-7 and MDA-MB-231 cancer cells, compared to untreated cells; bcl2 levels were notably (*p* < 0.05, *p* < 0.01) decreased in MCF-7 and MDA-MB-231, respectively, compared to untreated cells. Regarding c-PARP protein levels, treatment with azilsartan significantly (*p* < 0.01, *p* < 0.001) increased its protein levels in MCF-7 and MDA-MB-231 cancer cells, respectively, when compared to untreated cells.

### 2.7. Expression of NF-kB, TWIST, SNAIL, SLUG, Bax, and Bcl2 Genes

**Figure 7:**
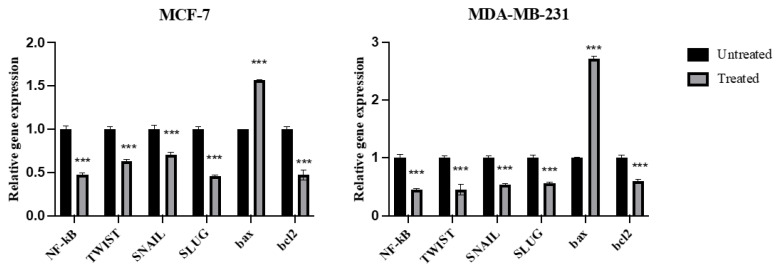
Effect of azilsartan on the expression of *NF-kB, TWIST, SNAIL, SLUG, bax* and *bcl2* genes in MCF-7 and MDA-MB-231 breast cancer cell lines. Relative gene expression in both cancer cells treated with the IC50 concentration of azilsartan compared to untreated cells. Expression was normalized to the corresponding *GAPDH* gene expression. Bars represent mean ± SD, *n* = 3. Significant difference was analyzed by two-way ANOVA, where *** *p* < 0.001, compared to untreated cells.

As shown in Figure 7, azilsartan significantly (*p* < 0.001) decreased the expression of NF-kB, twist family bHLH transcription factor 1 (TWIST), snail family zinc finger 1 (SNAIL), snail family zinc finger 2 (SLUG) and bcl2 genes, when compared to untreated cells, in MCF-7 and MDA-MB-231 breast cancer cells. In addition, it significantly (*p* < 0.001) increased the expression of bax gene in both cancer cell lines, when compared to untreated cells.

### 2.8. Expression of MMP9, p-STAT3, JAK2, IL6 and Cleaved Caspase Proteins

**Figure 8:**
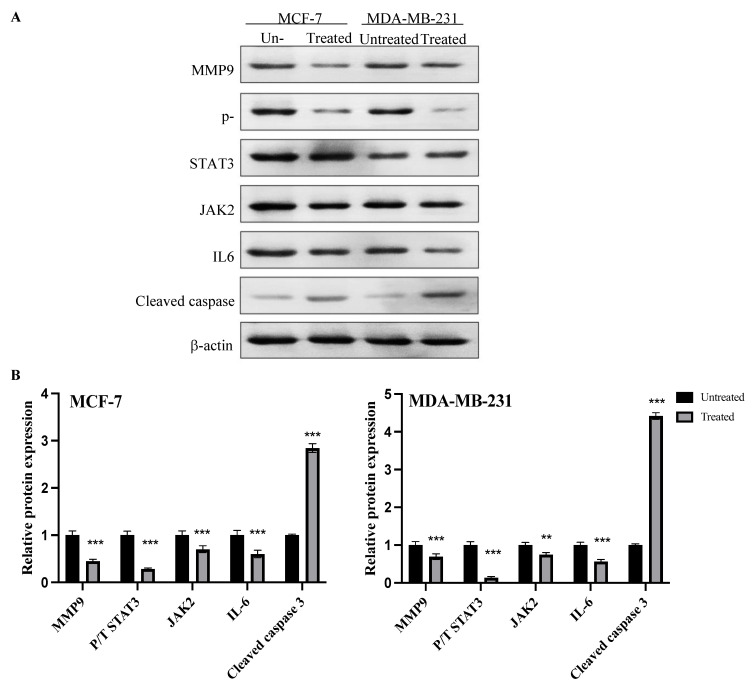
Effect of azilsartan on the expression of MMP9, phosphorylated STAT3, total STAT3, JAK2, IL6 and cleaved caspase proteins in MCF-7 and MDA-MB-231 cancer cell lines. (**A**) Representative western blots of MMP9, phosphorylated STAT3, total STAT3, JAK2, IL6 and cleaved caspase proteins in MCF-7 and MDA-MB-231 cells treated with the IC_50_ concentration of azilsartan for 48 h. β-actin was used as internal loading control. (**B**) Expression of proteins in MCF-7 and MDA-MB-231 treated cells were expressed relative to untreated cells after normalization to the corresponding β-actin protein expression. Bars represent mean ± SD, *n* = 3. Significant difference was analyzed by two-way ANOVA test, where ** *p* < 0.01, *** *p* < 0.001, compared to untreated cells.

Expression of matrix metalloproteinase (MMP9), phosphorylated STAT3, total STAT3, JAK2, IL6 and cleaved caspase proteins in MCF-7 and MDA-MB-231 cancer cells before and after treatment with the IC50 of azilsartan for 48 h is shown in Figure 8. After normalization to the internal control β-actin, MMP9, phosphorylated/total STAT3 and IL6 proteins expression was significantly (*p* < 0.001) decreased and the expression of cleaved caspase 3 protein was significantly (*p* < 0.001) increased in both MCF-7 and MDA-MB-231 cancer cells after treatment with azilsartan, when compared to the untreated cells. The treatment with azilsartan significantly (*p* < 0.001 and *p* < 0.01) decreased the expression of JAK2 protein in MCF-7 and MDA-MB-231 cancer cell lines, respectively, when compared to the untreated cells.

### 2.9. Effect of Azilsartan on TNFα-Induced NF-kB Activation

**Figure 9:**
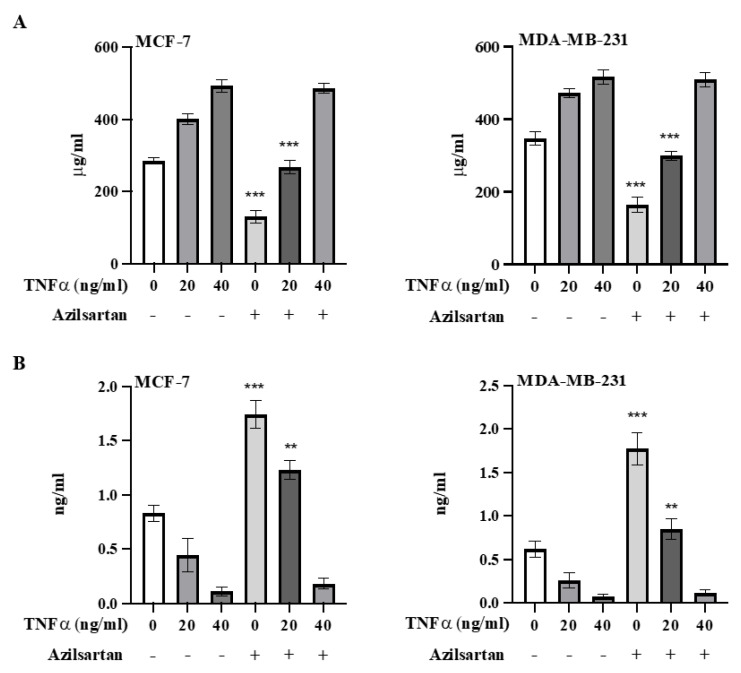
Effect of azilsartan on TNFα-induced NF-kB activation. The levels of NF-kB p65 (**A**) and cleaved caspase 3 (**B**) proteins in non-stimulated and TNFα (20 and 40 ng/mL)-stimulated MCF-7 and MDA-MB-231 cancer cell lines were measured. Bars represent mean ± SD, *n* = 3. Significant difference was analyzed by student *t* test, where ** *p* < 0.01, *** *p* < 0.001, compared to the corresponding azilsartan-untreated cells.

To confirm the role of azilsartan in targeting NF-kB pathway and initiating apoptosis in MCF-7 and MDA-MB-231 breast cancer cells, cells were stimulated with TNFα after the treatment with the IC50 of azilsartan and the levels of NF-kB p65 and cleaved caspase 3 proteins were measured. As shown in Figure 9A, azilsartan significantly (*p* < 0.001) decreased NF-kB protein levels in non-stimulated and 20 ng/mL TNFα-stimulated MCF-7 and MDA-MB-231 breast cancer cells. Cells stimulated with 40 ng/mL TNFα showed no significant difference in NF-kB protein levels when treated with azilsartan, compared to the corresponding untreated cells.

In addition, in both cancer cell lines, azilsartan exerted a significant (*p* < 0.001, *p* < 0.01) increase in the levels of cleaved caspase 3 protein in non-stimulated and 20 ng/mL TNFα-stimulated cells, respectively, compared to the corresponding untreated cells. However, when both cancer cell lines were stimulated with 40 ng/mL TNFα, azilsartan showed no significant change in cleaved caspase 3 protein levels, compared to the corresponding untreated cells, as shown in Figure 9B.

### 2.10. Effect of Azilsartan on the In Vivo Tumor Metastatic Activity

**Figure 10:**
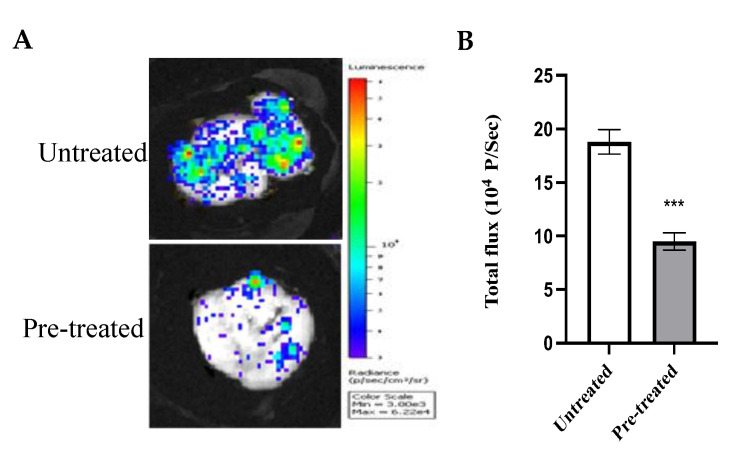
Effect of azilsartan on the in vivo tumor metastatic activity of MDA-MB-231/Luc cancer cells. (**A**) Bioluminescent images for lungs of animals injected with cancer cells untreated or pre-treated with azilsartan. (**B**) The bioluminescent intensities. Bars represent mean ± SD, *n* = 6. Significant difference was analyzed by student *t* test, where *** *p* < 0.001, compared to intensities of the animals injected with untreated cells.

To further elucidate the anti-metastatic activity of azilsartan in vivo, a stable luciferase-expressing reporter breast cancer cell line, MDA-MBA-231/Luc, was utilized. Untreated and pre-treated MDA-MB-231/Luc cells with the IC50 of azilsartan were injected into animals. After 5 days, lung tissues were collected and visualized, as shown in Figure 10. Animals injected with azilsartan-pre-treated cells showed significant (*p* < 0.001) inhibition of metastatic cancer cells, when compared to animals injected with untreated cells.

## 3. Discussion

Metastatic breast cancer occurs when cancer cells detach from the primary tumor and invade the adjacent tissues to reach the blood stream and colonize at distant organs [31,32]. The development of distant metastasis is a crucial event that limits the survival of breast cancer patients [33]. To date, the EMT phenomenon, the loss of epithelial characteristics and the gain of mesenchymal features, is the most reasonable explanation of distant metastasis of epithelial cancers, including breast cancer [34,35,36]. The EMT transition enables breast cancer cells to exhibit a self-renewal characteristic, and to enhance cell motility, which results in the occurrence of metastatic colonies at distant sites [37,38]. Therefore, inhibition of the metastatic ability and the EMT transition of breast cancer cells are required.

Apart from the role of Ang II in regulating blood pressure, Ang II majorly contributes to inflammation, fibrosis, angiogenesis, migration, invasion and cancer progression [39,40]. Rodrigues-Ferreira et al. indicated that direct exposure of breast cancer cells to Ang II promoted trans-endothelial motility and migration and accelerated metastatic progression [33]. Thus, the use of ARB could be a potential approach in breast cancer therapies.

Another critical player of breast cancer metastasis is STAT3 signaling pathway. Ang II activates NF-kB which, in turn, increases the expression of IL-6, leading to activation of the JAK2/STAT3 signaling pathway [18]. It was reported that the activation of IL-6/JAK2/STAT3 pathway promotes proliferation, invasion, metastasis and angiogenesis, and inhibits apoptosis in breast cancers [41,42]. Therefore, targeting Angiotensin initiation of NF-kB/IL-6/JAK2/STAT3 pathway could be a beneficial strategy in breast cancer therapies.

Recently, repurposing drugs has received great attention [43,44,45,46]. Azilsartan is a potent ARB and FDA approved for the management of hypertension [47]. Azilsartan selectively binds to angiotensin II type 1 (AT1) receptors, which are highly expressed in vascular tissues, causing significant prevention of vasoconstriction and providing a highly effective candidate in treating blood pressure [48]. Recently, it was reported that azilsartan exerted a gastroprotective effect in rats through its anti-inflammatory and antioxidant activities and by restoring the gastrin and hydroxyproline levels [49]. Furthermore, azilsartan induced ROS production, cytochrome c release and cytotoxicity in liver hepatocellular carcinoma cell line (HepG2) and human lung cancer cell line (A549) [6,50]. The present study, to our knowledge, is the first one which showed the new potential effect of azilsartan on the proliferation, colony formation and migration abilities of breast cancer cells, explaining, on molecular levels, the molecular mechanism underlying this new potential effect.

MCF-7 and MDA-MB-231 breast cancer cell lines were treated with azilsartan at different concentrations. Our findings revealed that azilsartan showed significant inhibition of cell survival and induced apoptosis and cell cycle arrest in both cancer cell lines. However, it reduced the viability of MCF 10A breast epithelial normal cells at higher concentrations, ensuring its safety. In addition, we further examined the effect of azilsartan on the colony formation and migration abilities of these cancer cells. The present study showed that azilsartan suppressed the in vitro colony forming efficiency and cell migration ability in a significant manner in both cancer cell lines. In addition, the present study revealed that it reduced the in vivo lung colonization of MDA-MB-231 cancer cells which further confirmed our hypothesis about the anti-metastatic activity of azilsartan against breast cancer cells.

Furthermore, to check the activity of azilsartan in both cancer cell lines at the molecular level, NF-kB/IL-6/JAK2/STAT3 signaling pathway was investigated. Our data showed that azilsartan treatment reduced the expression of *NF-kB* mRNA, which is involved in many cellular pathways in malignant tumors, and decreased the expression of IL-6, JAK2 and STAT3 proteins, which promote the proliferation, metastasis, invasion and angiogenesis in breast cancers, resulting in the suppression of this NF-kB/IL-6/JAK2/STAT3 signaling pathway.

Many studies documented that STAT-3-mediated breast cancer metastasis happens through the upregulation of MMP9, TWIST, SNAIL and SLUG expression [42,51,52]. Targeting MMP9 can reduce breast cancer progression and modulates EMT genes [53,54]. In the present study, azilsartan was found to inhibit the expression of MMP9 protein in both MCF-7 and MDA-MB-231 breast cancer cell lines.

Furthermore, EMT markers, including TWIST, SNAIL and SLUG, were investigated in the present study. SLUG is a TF which regulates the expression of EMT genes. The expression of SLUG inhibits E-cadherin expression, leading to the suppression of intercellular adhesion and the initiation of cell motility properties [55]. TWIST is another TF that acts as a predominant regulator of EMT and is associated with cancer stem cell phenotyping [56,57,58]. Moreover, TWIST was shown to inhibit p53 pathways, resulting in antiapoptotic actions [59]. SNAIL is also a TF which controls EMT during embryogenesis and tumor progression [60]. Data of the present study revealed that the expression of *TWIST, SNAIL* and *SLUG* genes were significantly reduced in both cancer cell lines after the treatment with azilsartan, which may suggest the suppression of EMT in breast cancer cells by azilsartan.

**Figure 11:**
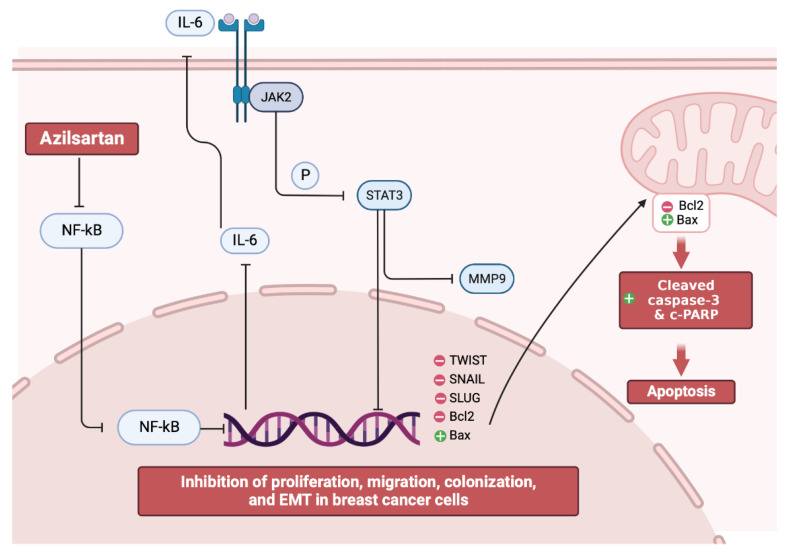
Illustrative figure summarizes the molecular mechanism of the anti-proliferative, EMT inhibition, and apoptotic activities of azilsartan against breast cancer cells.

Interestingly, it was reported that the activated IL-6/JAK2/STAT3 pathway can inhibit bax/bcl2 related caspase-dependent apoptosis, leading to the promotion of proliferation and metastasis of cancer cells [61]. Thus, targeting apoptosis became a therapeutic approach in cancer [62,63,64]. In the present study, the mRNA and protein levels of *bax* and *bcl2* in addition to the levels of cleaved caspase 3 and c-PARP proteins were examined. Our findings revealed that the expression of bax (a pro-apoptotic factor), cleaved caspase 3 and c-PARP proteins were substantially increased, while the expression of bcl2 (an anti-apoptotic factor) was decreased in both MCF-7 and MDA-MB-231 breast cancer cell lines by azilsartan leading to the induction of apoptosis, as summarized in Figure 11, which was also confirmed by the flow cytometric analysis.

To confirm the role of azilsartan in targeting and inhibiting NF-kB pathway with subsequent initiation of apoptosis, MCF-7 and MDA-MB-231 cancer cells were stimulated with the inflammatory cytokine TNFα, which was reported to activate NF-kB pathway, resulting in further invasion and metastasis of breast cancer cells [65]. Interestingly, at 20 ng/mL TNFα stimulation of MCF-7 and MDA-MB-231 cancer cells, azilsartan still showed notable reduction in NF-kB p65 protein levels and substantial elevation in cleaved caspase 3 protein, which confirms the targeted activity of azilsartan on NF-kB pathway to initiate apoptosis in MCF-7 and MDA-MB-231 breast cancer cell lines.

However, further in vivo experimental studies are required to emphasize the mechanistic activity of azilsartan as a promising anticancer agent against breast cancer cells.

## 4. Materials and Methods

### 4.1. Cell Culture

MCF-7, MDA-MB-231 and MCF 10A cell lines were obtained from American type culture collection (ATCC, Manassas, VA, USA). Fresh Dulbecco’s Modified Eagle’s Medium (DMEM, Sigma-Aldrich, Inc., St Louis, MO, USA) was used as a culture medium, augmented with 10% fetal bovine serum (FBS, Biosolutions International, Melbourne, Australia), 1% penicillin-streptomycin mixture (Invitrogen, Grand Island, NY, USA) and 1% L-glutamine (Sigma-Aldrich, Inc., St Louis, MO, USA) in a humidified 5% CO_2_ atmosphere at 3 °C.

### 4.2. Cell Viability

Cell viability assay was achieved using MTT reagent [3-(4, 5-dimethyl thiazol-2yl)-2, 5-diphenyltetrazolium bromide]. MCF-7, MDA-MB-231 or MCF 10A cells (10^4^ cells per well) were seeded in triplicate in 96-well plates and allowed to grow in fresh DMEM medium for 24 h. Then, medium was changed with fresh DMEM containing different concentrations (7.81, 15.61, 31.25, 62.5, 125, 250, 500 and 1000 μg/mL) of azilsartan (Sigma-Aldrich, Inc., St Louis, MO, USA). After 48 h, 10 μL of MTT (5 μg/mL) was added per well and incubated in the dark for 3 h at 37 °C. To dissolve the Formazan crystals that were formed, 100 μL of DMSO was used, and absorbance was measured using an ELISA reader at 570 nM [66]. The IC_50_ of azilsartan was calculated for each cell line using Graph Pad Prism-9 software for macOS (GraphPad version 9.4.1 (458), La Jolla, CA, USA).

### 4.3. Annexin V Assay

By flow cytometric analysis, Annexin V-FITC Apoptosis Detection Kit (Sigma-Aldrich, Inc., St Louis, MO, USA) was used to detect apoptosis, according to the manufacturer’s instructions. The combination of annexin V and propidium iodide allowed discrimination between live cells (annexin V negative, PI negative), early apoptotic cells (annexin V positive, PI negative), late apoptotic (annexin V positive, PI positive) and necrotic cells (annexin V negative, PI positive). Using the IC_50_ of azilsartan, the cancer cells were treated for 48 h. Cells were suspended in 1X Binding Buffer at a concentration of ≈1 × 10^6^ cells/mL. Then, 5 μL of FITC-Conjugated Annexin V and 10 μL of propidium iodide solution were added to 500 μL of cell suspensions. The cells were incubated at room temperature for 10 min in the dark. Cell fluorescence was determined immediately with a flow cytometer (Becton Dickinson, Franklin Lakes, NJ, USA), counting at least 10^4^ cells. Dot plots were obtained, and percentage of apoptotic cells was estimated.

### 4.4. Cell Cycle Analysis

To evaluate the cell cycle distribution, propidium iodide staining was used. The experiment was done in triplicate. A total of 1 × 10^6^ cells of both untreated and treated cancer cells with the IC_50_ of azilsartan for 48 h were collected by centrifugation, washed with phosphate buffer saline (PBS, pH = 7.4, Sigma-Aldrich, Inc., St Louis, MO, USA) and fixed with ice cold 66% ethanol. The fixed cells were washed with PBS and resuspended for 30 min in 1 mg/mL RNAse. Propidium iodide (50 μg/mL) was used to label the intracellular DNA by incubating the cells for at least 20 min at 4 °C in the dark. Then, samples were analyzed using flow cytometry.

### 4.5. Colony Formation Assay

Cells were seeded in triplicate in 24-well plates at 100 cells/well and allowed to attach for 24 h in fresh DMEM medium. Then, the medium was changed to DMEM containing IC_50_ of azilsartan, and cells were incubated for 48 h. Then, the cells were washed with PBS twice and allowed to grow in fresh DMEM medium for 14 days at 37 °C under humidified 5% CO_2_ conditions. The medium was changed every 2–3 days. Then, cells were fixed in 4% formaldehyde after washing with PBS and stained for 15 min with crystal violet. Number of colonies was estimated by manual counting using stereo microscope (Jenco^™^ stereo microscopes, GL series, Sigma Aldrich Co., St Louis, MO, USA). Colony forming efficiency (CFE) was assessed by the following equation [67]:

(1) $$\mathrm{CFE}=\frac{numberof\;colonies\;formed}{number\;of\;cells\;plated}\times 100\%$$

### 4.6. Cell Migration Assay

Cells (1 × 10^6^ cells) were seeded in triplicate in 6-well plate culture dish in DMEM medium and incubated at 37 °C in humidified 5% CO_2_ conditions until confluent. Then, a wound was created in the formed monolayers using 200 µL tips. The scratch was done under an angle of around 30 degrees to keep the scratch width limited. Cells were washed with PBS, treated with the IC_50_ of azilsartan in DMEM, and incubated for 48 h. Using a live-cell imaging system, photos were captured using the cytosmart system. Using ImageJ, estimation of the open scratch area was performed at different points, covering all the distances that cells had migrated, and data were analyzed using GraphPad Prism-9 software for macOS (GraphPad version 9.4.1 (458), La Jolla, CA, USA).

### 4.7. Measurement the Levels of Bax, Bcl2, Cleaved PARP Proteins

To measure the levels of bax, bcl2 and c-PARP proteins, bax ELISA kit (#MBS701787, MyBioSource, CA, USA), bcl2 ELISA kit (#MBS701787, MyBioSource) and c-PARP ELISA kit (#KHO0741, Life Technologies Ltd., Paisley, UK) were utilized. MCF-7 and MDA-MB-231 cancer cells were treated with the IC50 of azilsartan for 48 h. Then, cells were harvested, followed by the measurement of the levels of bax, bcl2 and c-PARP proteins according to the manufacturer’s instructions. Standard curves were obtained, and the absorbance was measured at 450 nm. The experiment was performed in triplicate.

### 4.8. RNA Isolation and Real-Time PCR Assay

The expression of the *NF-kB, TWIST, SNAIL, SLUG, bax* and *bcl2* genes were assessed by real-time PCR. Cancer cells were treated with the IC_50_ of azilsartan for 48 h. Then, total RNA was extracted from the cells according to the Qiagen RNA extraction kit (Hilden, Germany) instructions. Quantification of mRNA was achieved by utilizing the Rotor-Gene 6000 Series Software 1.7. *Glyceraldehyde 3-phosphate dehydrogenase* (*GAPDH)* was used as internal control [68]. The sequences of the primers, obtained from National Center for Biotechnology Information (NCBI), are mentioned in Table 1. RT-PCR reactions, performed using the Qiagen one step RT-PCR (Qiagen), contained 100 ng of total RNA, 1× buffer, 0.6 μM forward and reverse primers, 400 μM each of dNTP and 2 μL enzyme mix. The conditions were as following: 35 cycles of 25 sec denaturation step at 95 °C, 30 Sec primer annealing at 58 °C and 20 Sec polymerization steps at 72 °C. Triplicate RT-PCR reactions were performed for each sample. Cycle threshold (Ct) was determined for each sample, and the average Ct was calculated. To exclude the generation of non-specific compounds and to characterize the obtained amplified mixture with the avoidance of contamination, a melting curve analysis was achieved between 60–95 °C, at 1 °C intervals with the Rotor-Gene 6000 Series Software 1.7 using the SYBR Green fluorescent dye. After normalization to the internal control *GAPDH* expression, the target gene expression in the treated cells relative to the untreated ones was calculated.

### 4.9. Analysis of Protein Expression via Western Blotting

To examine the expression levels of MMP9, cleaved caspase 3, phosphorylated STAT3, total STAT3, JAK2 and IL6 proteins, sodium dodecyl sulphate–polyacrylamide gel electrophoresis (SDS-PAGE) analysis was performed. Cells were treated with the IC_50_ of azilsartan for 48 h. Then, the cells were collected, and protein extraction was performed in RIPA lysis buffer, containing 50 mM Tris–Cl, pH 7.5; 0.1% SDS, 150 mM NaCl, 0.5% sodium deoxycholate, 1 mM PMSF and 1% Nonidet P-40, supplemented with the complete protease inhibitor cocktail (Roche, Mannheim, Germany). The Bradford method was used to determine the protein concentration [69]. Cell lysates containing 30 μg protein were separated by SDS-PAGE (15% acrylamide), transferred to a Hybond™ nylon membrane (GE Healthcare) and incubated for 1 h at room temperature in Blocking Solution. Membranes were incubated overnight at 4 °C with MMP9, cleaved caspase, phosphorylated STAT3, total STAT3, JAK2 and IL6 antibodies (New England Biolabs, Ipswich, MA, USA) diluted (1:1000) with PBS. Then, membranes were washed for 30–60 min and incubated at room temperature for 1 h with the HRP-conjugated secondary antibody (New England Biolabs, Ipswich, MA, USA) diluted (1:1000) in PBS [70]. According to the manufacturer’s instructions, immunoreactive proteins were detected using an enhanced chemiluminescence kit (GE Healthcare, Little Chalfont, UK) by a luminescent image analyzer (LAS-4000, Fujifilm Co., Tokyo, Japan). Antibody against β-actin (New England Biolabs) (1:1000) was used to detect β-actin, which was used as a loading control. Electrophoresis and electroblotting, using a discontinuous buffer system, were carried out in a Bio-Rad Trans-Blot SD Cell apparatus (Bio-Rad, Hercules, CA, USA). Densitometric analysis was then performed by using The Image Processing and Analysis Java (ImageJ) program. Data were normalized to β-actin levels.

### 4.10. Evaluation of Azilsartan Activity on TNFα-Induced NF-kB Activation in MCF-7 and MDA-MB-231 Cancer Cell Lines

To confirm the role of azilsartan in targeting and inhibiting the activated NF-kB pathway in MCF-7 and MDA-MB-231 breast cancer cells, TNFα (#H8916, Sigma Aldrich, St Louis, MO, USA) was used to induce NF-kB in both MCF-7 and MDA-MB-231 cancer cells, as previously described in studies [65,71]. At first, cancer cells were treated with the IC50 of azilsartan for 48 h, followed by stimulation with 20 and 40 ng/mL of TNFα for 15 min. Cells were fixed with methanol, followed by measurement of the levels of NF-kB p65 and cleaved caspase 3 proteins utilizing NF-kB ELISA kit (#ab176648, Abcam, MA, USA) and cleaved caspase 3 ELISA kit (#KHO1091, Invitrogen, Grand Island, NY, USA) according to manufacturer’s instructions. The experiments were performed in triplicate.

### 4.11. The In Vivo Tumor Metastasis Assay

Twelve BALB/c mice were obtained from the animal house of Faculty of Pharmacy, Minia University. Mice were divided in two groups and maintained at a 12-h light/dark cycle in a temperature- and pressure-controlled animal room. Animal care and study protocols were followed according to the guidelines established by The Experimental Animal Center and Research Ethics Committee, Minia University (ES18/2021).

MDA-MB-231/Luc cancer cells, obtained from Cell Biolabs Incorporated (#AKR-231, Cell Biolabs, Inc., San Diego, CA, USA), were cultured with or without the IC50 of azilsartan for 24 h in complete DMEM media. Then, animals were intravenously injected via tail with 1 × 10^6^ cells in 0.1 mL PBS/mice. Five days after cancer cells injection, animals were intraperitonially injected with 200 μL firefly D-luciferin (#L9504, Sigma Aldrich Inc., St Louis, MO, USA) (10 mg/mL). After 10 min, animals were anaesthetized with xylazine (3 mg/mL) and ketamine (7 mg/mL) in PBS, followed by sacrification. Lung tissues were collected for bioluminescence visualization using the in vivo imaging system (Xenogen IVIS-200, Caliper Life Science, Hopkinton, MA, USA).

### 4.12. Statistical Analysis

At least three independent experiments were used to obtain the results. Data were expressed as mean ± standard deviation. Student’s *t*-test was used to analyze differences after one- or two-way analysis of variance (ANOVA), with the use of GraphPad Prism 9 statistical software for macOS (GraphPad version 9.4.1 (458), La Jolla, CA, USA) and Microsoft Excel software (Microsoft version 16.66.1, Redwood, WA, USA). Differences were considered significant when the probability values (*p*) were less than 0.05.

## 5. Conclusions

The present study revealed, for the first time, the anti-proliferative, anti-migration, EMT inhibition and apoptotic activities of azilsartan, which is an ARB, against MCF-7 and MDA-MB-231 breast cancer cell lines through modulating the NF-kB/IL-6/JAK2/STAT3/MMP9, TWIST/SNAIL/SLUG and bax/bcl2 mediated caspase-dependent signaling pathways.

## Figures and Tables

**Table 1:** Sequences of the primers.

Primer	Sequence
*TNF* *α*	Forward: 5′-CGCTTAGGAGGGAGAGCCCA-3′ Reverse: 5′-TGCCATTCTGAAGCCGGGTG-3′
*NF-kB*	Forward: 5′-ATGTCCGCGTCCCACTAGCA-3′ Reverse: 5′-GCCCCACGCCCTGTTTCTTT-3′
*TWIST*	Forward: 5′-CGGCCTAGCGAGTGGTTCTT-3′ Reverse: 5′-AGGAAAGAGCGCGGCATAGT-3′
*SNAIL*	Forward:5′-ATCTGCGGCAAGGCGTTTTCCA-3′ Reverse: 5′-GAGCCCTCAGATTTGACCTGTC-3‘
*SLUG*	Forward: 5′-GTTTCA TCC AGG ATC GAG CAG-3′ Reverse: 5′-CATCTT CTT CCA GAT GGT GA-3′
*bax*	Forward: 5′-CCTGTG GAT GAC TGA GTA CC-3′ Reverse: 5′-GAGACA GCC AGG AGA AAT CA-3′
*Bcl2*	Forward: 5′-CCCAGAAGACAGTGGACGGG-3′ Reverse: 5′-CGACAGACACATCCGGGGTT-3′
*GAPDH*	Forward: 5′-CGCTTAGGAGGGAGAGCCCA-3′ Reverse: 5′-TGCCATTCTGAAGCCGGGTG-3′

## Data Availability

All data are fully available and included in the manuscript.
